# Changes in Insurance Coverage Continuity After Affordable Care Act Expansion of Medicaid Eligibility for Young Adults With Low Income in Massachusetts

**DOI:** 10.1001/jamahealthforum.2022.1996

**Published:** 2022-07-15

**Authors:** Vicki Fung, Zhiyou Yang, Benjamin L. Cook, John Hsu, Joseph P. Newhouse

**Affiliations:** 1 Mongan Institute, Massachusetts General Hospital, Boston; 2 Department of Medicine, Harvard Medical School, Boston, Massachusetts; 3 Health Equity Research Lab, Cambridge Health Alliance, Cambridge, Massachusetts; 4 Department of Psychiatry, Harvard Medical School, Boston, Massachusetts; 5 Department of Health Care Policy, Harvard Medical School, Boston, Massachusetts; 6 Department of Health Policy and Management, Harvard T.H. Chan School of Public Health, Boston, Massachusetts; 7 Harvard Kennedy School, Cambridge, Massachusetts; 8 National Bureau of Economic Research, Cambridge, Massachusetts

## Abstract

**Importance:**

Young adults historically have had the highest uninsured rates among all age groups. In 2014, in addition to Medicaid expansion for adults with low income (≤133% of the federal poverty level [FPL]) through the Patient Protection and Affordable Care Act, Massachusetts also extended eligibility for children (≤150% FPL) to beneficiaries aged 19 to 20 years.

**Objective:**

To examine changes in insurance coverage continuity for Medicaid enrollees who turned age 19 years before and after eligibility policy changes.

**Design, Setting, and Participants:**

This cohort study used data from the Massachusetts All-Payer Claims Database (2012 to 2016) to compare coverage for Medicaid beneficiaries turning age 19 years before and after Medicaid expansion. Monthly coverage was examined for each cohort for 3 years as beneficiaries aged from 18 and 19 years to 19 and 20 years to 20 and 21 years. Analyses were performed between November 1, 2020, and May 12, 2022.

**Main Outcomes and Measures:**

In each year, the likelihood of being uninsured or having Medicaid, employer-sponsored insurance, or individual commercial coverage for 3 or more months was examined along with the likelihood of having continuous Medicaid enrollment for 12 or more and 24 or more months. Multivariable linear probability models were used to compare the likelihood of these outcomes for those in the postexpansion vs preexpansion cohorts, adjusting for sex, comorbidity levels, neighborhood socioeconomic status, and neighborhood race and ethnicity.

**Results:**

A total of 41 247 young adults turning age 18 to 19 years in the baseline year (20 876 [50.6%] men) were included in the study, with 20 777 in the preexpansion cohort and 20 470 in the postexpansion cohort. Enrollees who turned age 19 years after vs before the Medicaid eligibility expansion were less likely to have 3 or more uninsured months at ages 18 to 19 years (4.4% [n = 891] vs 22.9% [n = 4750]; adjusted difference, −18.4 [95% CI, −19.0 to −17.7] percentage points) and 19 to 20 years (13.2% [n = 2702] vs 35.8% [n = 7447]; adjusted difference, −22.4 [95% CI, −23.2 to −21.6] percentage points) and more likely to have continuous insurance coverage for 12 or more months (94.1% [n = 19 272] vs 63.7% [n = 13 234]; adjusted difference, 30.5 [95% CI, 29.7-31.2] percentage points) or 24 or more months (77.5% [n = 15 868] vs 44.4% [n = 9221]; adjusted difference, 33.0 [95% CI, 32.1-33.9] percentage points). Differences in the likelihood of having 3 or more uninsured months diminished at ages 20 to 21 years, when both groups had access to Medicaid (ie, in calendar years 2014 for the preexpansion cohort and 2016 for the postexpansion cohort).

**Conclusions and Relevance:**

In this cohort study of young adults in Massachusetts, the combination of expanding Medicaid to lower-income adults and increasing the age threshold for child Medicaid eligibility was associated with reduced likelihood of becoming uninsured among Medicaid enrollees entering adulthood.

## Key Points

**Question:**

Did Medicaid expansion in Massachusetts change coverage continuity for child Medicaid enrollees entering young adulthood?

**Findings:**

In this cohort study of 41 247 young adults, Medicaid enrollees who turned 19 after vs before Medicaid expansion were significantly less likely to have 3 or more months without insurance coverage at ages 18 to 19 and 19 to 20 years and more likely to have continuous Medicaid coverage for 24 or more months.

**Meaning:**

Federal and state Medicaid expansions were associated with reductions in insurance disruptions and coverage gaps among child Medicaid enrollees entering young adulthood.

## Teaser

This cohort study examines insurance coverage changes among young adults in Massachusetts after Medicaid eligibility was expanded.

## Introduction

Maintaining insurance coverage is challenging for young adults. Those in low-income households may age out of eligibility as children for public coverage, such as Medicaid or the Children’s Health Insurance Program, at age 19 years. Some young adults also face challenges accessing employer-sponsored coverage as they enter the workforce.^1^ The Patient Protection and Affordable Care Act (ACA) included numerous reforms that were intended to be and have been associated with coverage gains for young adults.^1,2^ Since 2014, 37 states have adopted Medicaid expansion for childless adults with incomes up to 133% of the federal poverty level (FPL). Since 2010, the ACA has also allowed dependents up to age 26 years to remain covered by parents’ private plans.^3,4,5^ The ACA reforms to the individual insurance market, including the provision of low-income subsidies, also increased access to commercial coverage for young adults who are not eligible for Medicaid or do not have employer-sponsored insurance.

Nevertheless, young adults continue to have the highest uninsured rates across all age groups.^6^ The largest increase in the proportion of uninsured occurs between ages 18 and 19 years, when age-based eligibility for children’s Medicaid or Children’s Health Insurance Program ends in most states. In 2019, 14.3% of individuals aged 19 years were uninsured compared with 4.8% of those aged 18 years.^6^ However, there is large variation across states in uninsured rates among young adults, even among states that expanded Medicaid eligibility. In 2019, Massachusetts had the lowest uninsured rate among individuals aged 19 to 25 years (4.9%) compared with individuals in the same age group in Alaska (20.0%), another expansion state.^6,7^ In Texas, a nonexpansion state, uninsured rates were as high as 30.0% among those aged 19 to 25 years in 2019.^7^

Maintaining insurance coverage during the transition to adulthood could be particularly important for individuals who experience mental illness because adolescence and young adulthood represent the peak ages of onset for those with lifetime diagnoses.^8,9^ Among adolescents and young adults with mental illness, increases in out-of-pocket costs for care and lack of insurance coverage have been associated with lower likelihood of receiving treatment.^10,11,12^ For individuals with serious mental illness, such as psychosis, early intervention has been associated with improvements in symptoms and quality of life, underscoring the importance of maintaining access to care throughout the transition from adolescence to young adulthood.^13^

In 2014, Massachusetts expanded Medicaid eligibility to childless adults with incomes up to 133% of the FPL. In contrast to many states, Massachusetts also expanded the age threshold for Medicaid eligibility as children to include individuals aged 19 to 20 years, allowing this group to qualify for Medicaid at a higher income level (150% of the FPL). In addition, Massachusetts implemented policies similar to the ACA individual insurance market reforms earlier with the state’s 2006 health insurance law. The aim of this study was to examine changes in insurance coverage among young adults overall and among those with mental health diagnoses who were enrolled in Medicaid as children and were entering young adulthood (ages 18-21 years) before and after the Medicaid expansion in 2014.

## Methods

### Data Source

The data source for this cohort study is the Massachusetts All-Payer Claims Database (APCD), release 6.0, which includes insurance enrollment and medical claims data from 2012 through 2016 from Medicare Advantage plans, employer-sponsored plans, individual commercial plans, and Medicaid, as well as a master patient index that links individuals across different insurance carriers.^14^ The APCD does not include coverage through workers’ compensation, TRICARE and the Veterans Health Administration, and Federal Employee Health Benefit plans. It also does not include individuals enrolled in traditional fee-for-service Medicare. Because of the Supreme Court ruling in *Gobeille v Liberty Mutual Insurance Co*, self-funded employer plans were not required to submit data to the APCD starting in 2016.^15^ This study was approved by the Mass General Brigham Institutional Review Board with waiver of written informed consent because the research involves no more than minimal risk to included persons, the waiver would not adversely affect the rights and welfare of the participants, and the research could not practicably be carried out without the waiver. This study followed the Strengthening the Reporting of Observational Studies in Epidemiology (STROBE) reporting guideline.

### Study Population

We identified 2 Medicaid cohorts to examine enrollment patterns, including Medicaid enrollees between ages 18 and 21 years who turned age 19 years before and after Medicaid expansion in 2014. The preexpansion cohort included Medicaid enrollees who were age 18 years as of January 2012 and turned 19 years in the preexpansion period. The postexpansion cohort included Medicaid enrollees who were age 18 years as of January 2014 and turned 19 years in the postexpansion period. For individuals in each of these cohorts, we examined insurance coverage in each month for 36 months. Specifically, the preexpansion cohort was followed from January 1, 2012, to December 31, 2014, meaning that the observation period included 2 preexpansion years, during which individuals turned age 19 years, and 1 year after expansion in 2014, when individuals in the cohort were 20 to 21 years old. The postexpansion cohort was followed from January 1, 2014, to December 31, 2016; thus, all observation months were after Medicaid expansion (eFigure in the Supplement). Because the APCD does not include birth dates, we ascertained age based on the age (in years) at the time of enrollment record submission (December 2012 and 2014).

We also assessed changes in insurance coverage for a subsample of enrollees with documented mental health conditions. This group included those with *International Classification of Diseases, Ninth Revision, Clinical Modification* codes for anxiety disorders; depression; attention-deficit/hyperactivity disorder, conduct disorders, and hyperkinetic syndrome; bipolar disorder; schizophrenia and other psychotic disorders; or personality disorders in the baseline year (2012 for the preexpansion and 2014 for the postexpansion cohort) using algorithms developed by the Centers for Medicare and Medicaid Services Chronic Conditions Data Warehouse.^16^

In 2014, because of difficulties implementing systems to identify applicants’ eligibility for the Massachusetts Health Connector (individual commercial insurance) programs, Medicaid, and the Health Safety Net, the state temporarily extended subsidized individual commercial insurance for enrolled members and enrolled new Health Connector applicants, who otherwise would have been eligible for Health Connector programs or the Health Safety Net, into temporary Medicaid coverage. The Health Safety Net is a program that provides financial relief for some health services for the uninsured and underinsured.^17^ The state began redetermination processes for those with temporary coverage in 2015.^18,19^

### Insurance Coverage Outcomes

For each individual, we classified monthly insurance coverage as Medicaid, employer-sponsored insurance, individual commercial insurance (eg, Commonwealth Care, ConnectorCare), other insurance, Health Safety Net, or uninsured based on the APCD member eligibility file.

We classified months without an enrollment record for any type of coverage (including the Health Safety Net) as being uninsured. Relying on enrollment records could misclassify individuals who move out of state and are no longer covered by entities submitting data to the APCD as uninsured. To guard against this concern, we focused on comparisons between the preexpansion and postexpansion cohorts because we did not expect rates of movement out of Massachusetts to differ for this age group across these years.

We examined the likelihood of being (1) uninsured for at least 3 months, (2) enrolled in Medicaid for at least 3 months, and (3) commercially insured for at least 3 months in each year of follow-up (ie, at ages 18-19 years, 19-20 years, and 20-21 years); coverage could be in nonconsecutive months. We chose a 3-month cutoff to represent a lower bound of insurance discontinuity because even relatively short periods of uninsurance have been found to be associated with barriers to health care.^20^ In sensitivity analyses, we also examined a 6-month cutoff. In addition, we examined the likelihood of having continuous insurance coverage (of any type) and continuous Medicaid coverage for 12 or more months and 24 or more months since January 2012 (preexpansion cohort) and 2014 (postexpansion cohort).

### Statistical Analysis

We described coverage patterns during January 2012 to December 2014 for the preexpansion cohort and January 2014 to December 2016 for the postexpansion cohort by assessing the proportion of each cohort with each type of insurance in each of the 36 months of follow-up.

Next, we used separate multivariable linear probability models to compare the likelihood of being uninsured, enrolled in Medicaid, or commercially insured for at least 3 months in each year of follow-up for the postexpansion vs preexpansion cohorts, adjusting for covariates, including sex, comorbidity levels, neighborhood socioeconomic status, and neighborhood race and ethnicity (data were obtained from the American Community Survey). We used similar models to compare the likelihood of having continuous coverage of any type or Medicaid coverage for 12 or more or 24 or more months. We defined comorbidity levels using the Pediatric Medical Complexity Algorithm based on baseline year claims to classify individuals as having complex chronic disease, noncomplex chronic disease, and no chronic disease.^21^ For neighborhood socioeconomic status, we linked zip codes with 5-year estimates from the American Community Survey and classified zip codes with more than 20% of residents below the FPL or more than 25% of adults aged 25 years or older with less than a high school level of educational attainment as low socioeconomic status based on previously validated thresholds.^22,23,24^ Because we did not have information on individuals’ races and ethnicities, we also adjusted for zip code–level distributions of race and ethnicity from the American Community Survey (percentage of Hispanic or Latino and Non-Hispanic or Non-Latino Asian, Black or African American, White, and other individuals [other races included American Indian or Alaska Native, Native Hawaiian or Other Pacific Islander, some other race, and 2 or more races]).

A small proportion of individuals had enrollment records for Medicaid and commercial coverage in the same month. In primary analyses, we considered these months as commercial coverage. In sensitivity analyses, we changed the classification to Medicaid coverage. Because some self-insured plans stopped submitting data to the APCD starting in 2016, we could overestimate uninsured rates in the postexpansion cohort. To address this concern, we conducted sensitivity analyses in which we excluded self-insured enrollment records from the analyses in all years.

Two-tailed tests were conducted using Stata, version 15 (StataCorp LLC), and a 2-sided *P* < .05 was the threshold for statistical significance. Analyses were performed between November 1, 2020, and May 12, 2022.

## Results

### Study Population Characteristics

A total of 41 247 young adults turning from age 18 to 19 years in the baseline year were included in the study (20 876 men [50.6%] and 20 295 [49.2%] women; data on sex were missing or conflicting for 76 [0.2%] individuals); 20 777 Medicaid enrollees aged 18 years in January 2012 were included in the preexpansion cohort and 20 470 Medicaid enrollees aged 18 years in January 2014 in the postexpansion cohort. Young adults in the postexpansion vs preexpansion cohort were similar with respect to sex and were less likely to live in low socioeconomic status zip codes but were more likely to have complex and noncomplex chronic disease (Table 1). Those in the postexpansion cohort had a higher mean number of months insured in the baseline year as enrollees were turning age 19 years (11.4 vs 10.0; *P* < .001).

**Table 1.** Study Population Baseline-Year Characteristics^a^

Characteristic	No. (%)		*P* value^b^
	Preexpansion cohort (n = 20 777)	Postexpansion cohort (n = 20 470)	
Sex^c^			
Female	10 298 (49.6)	9997 (48.8)	.12
Male	10 433 (50.2)	10 443 (51.0)	
Low SES zip code^d^	6159 (29.6)	5919 (28.9)	.005
Race and ethnicity, mean (SD)^e^			
Hispanic or Latino	18.5 (19.3)	18.9 (19.8)	.07
Non-Hispanic or non-Latino Asian	5.0 (6.1)	5.3 (6.4)	<.001
Non-Hispanic or non-Latino Black or African American	11.3 (15.8)	11.1 (15.6)	.15
Non-Hispanic or non-Latino White	62.2 (26.8)	61.5 (26.9)	.02
Non-Hispanic or non-Latino other^f^	3.0 (2.1)	3.2 (2.3)	<.001
Pediatric medical complexity			
Complex chronic disease	1863 (9.0)	2160 (10.6)	<.001
Noncomplex chronic disease	3206 (15.4)	3690 (18.0)	
No chronic disease	15 708 (75.6)	14 620 (71.4)	
Mental health diagnosis in baseline year^g^	3454 (16.6)	3855 (18.8)	<.001
Insured months in baseline year, mean (SD)	10.0 (2.9)	11.4 (2.0)	<.001

Abbreviation: SES, socioeconomic status.

^a^ The preexpansion cohort included individuals who were aged 18 years and enrolled in Medicaid in January 2012; these individuals turned age 19 years before Medicaid expansion in 2014. The postexpansion cohort included individuals who were aged 18 years and enrolled in Medicaid in January 2014; these individuals turned age 19 years after Medicaid expansion.

^b^ Robust SEs were used to calculate most *P* values from *t* tests; pediatric medical complexity used *P* value from a χ^2^ test.

^c^ A total of 0.2% (46) of the preexpansion and 0.1% (30) of the postexpansion cohort had missing or conflicting sex data.

^d^ Low SES zip codes included areas with more than 20% of households below the federal poverty level or more than 25% of residents aged 25 years or older with less than a high school level of educational attainment.

^e^ Race and ethnicity included the percentage of individuals of each race and ethnicity who lived in the individual’s zip code.

^f^ Other races included American Indian or Alaska Native, Native Hawaiian or Other Pacific Islander, some other race, and 2 or more races.

^g^ Mental health diagnoses included anxiety disorders; depression; attention-deficit/hyperactivity disorder, conduct disorders, and hyperkinetic syndrome; bipolar disorder; schizophrenia and other psychotic disorders; or personality disorders.

Among individuals with mental health diagnoses in the baseline year, those in the postexpansion vs preexpansion cohorts were similarly likely to live in low socioeconomic status zip codes and to have complex or noncomplex chronic disease (eTable 1 in the Supplement). Anxiety disorder diagnoses were more prevalent in the postexpansion vs preexpansion cohort (54.6% [n = 2104 of 3855] vs 50.3% [n = 1736 of 3454]; *P* < .001), but the prevalence of diagnoses for other mental health conditions was similar.

### Monthly Coverage Patterns

In both cohorts, the proportion of individuals with Medicaid coverage decreased as individuals aged from 18 to 21 years, with larger decreases in Medicaid coverage in the preexpansion cohort during the first 24 months of follow-up (ie, before 2014, Figure). For example the proportion decreased to 36.3% at the end of the first 24 months for the preexpansion cohort, compared with 69.8% for the postexpansion cohort. Among the preexpansion cohort, the proportion of individuals with Medicaid coverage increased after 2014 in individuals aged 20 to 21 years after this group became eligible for Medicaid owing to the expansion (36.3% in December 2013 vs 59.8% in January 2014) and the proportion of uninsured individuals decreased (27.1% in December 2013 vs 22.3% in January 2014).

**Figure.**
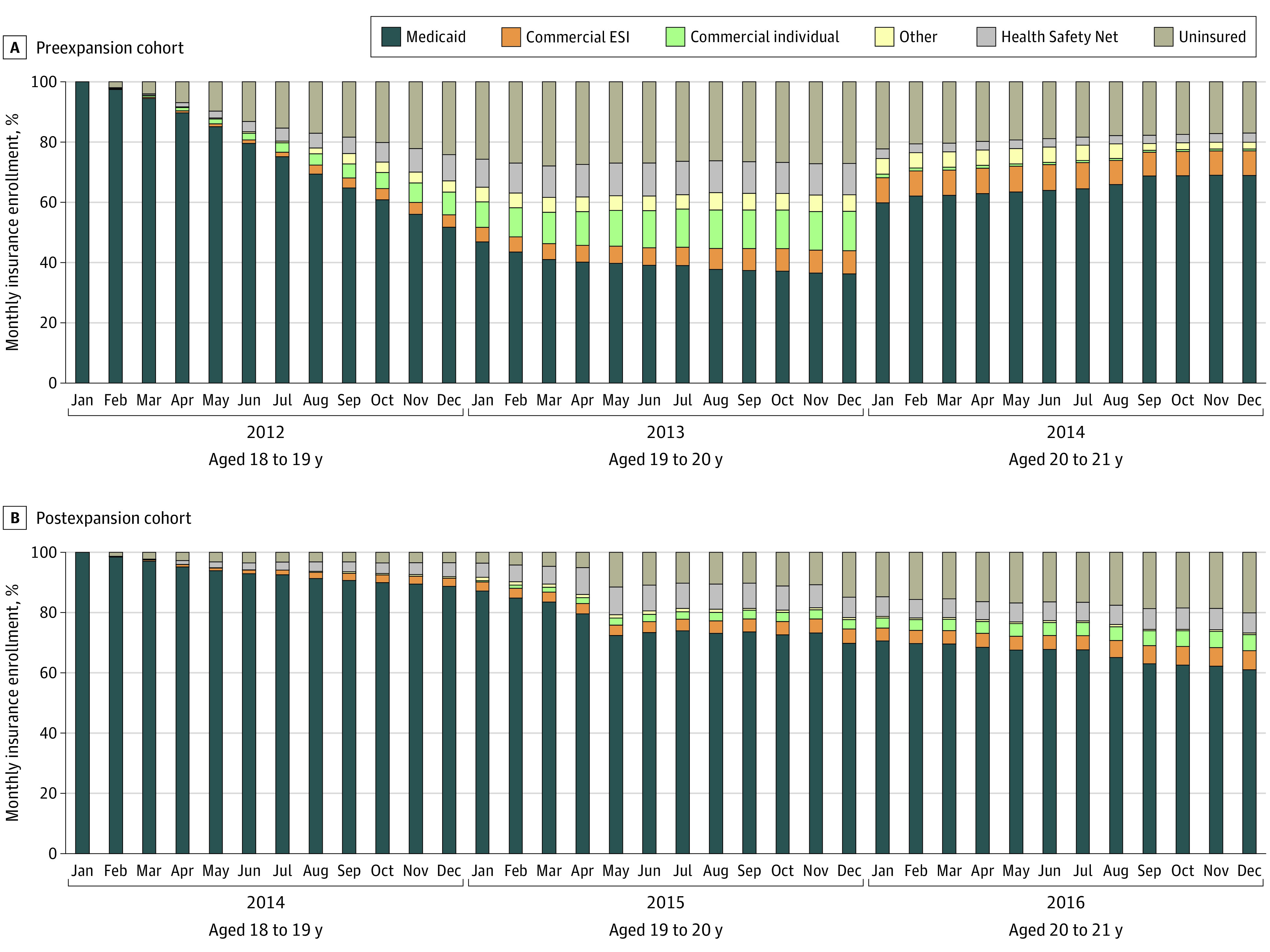
Monthly Enrollment Patterns for Medicaid Enrollees Age 18 Years in January 2012 and 2014 During the 36-Month Observation Period The preexpansion cohort included individuals who were age 18 years and enrolled in Medicaid in January 2012; these individuals turned age 19 years before Medicaid expansion in 2014. The postexpansion cohort included individuals who were age 18 years and enrolled in Medicaid in January 2014; these individuals turned age 19 years after Medicaid expansion. ESI indicates employer-sponsored insurance.

### Annual Coverage Differences

In multivariable analyses, individuals in the postexpansion cohort were less likely than those in the preexpansion cohort to be uninsured for 3 or more months at ages 18 to 19 years (4.4% [n = 891 of 20 470] vs 22.9% [n = 4750 of 20 777]; adjusted difference, −18.4 [95% CI, −19.0 to −17.7] percentage points) and at ages 19 to 20 years (13.2% [n = 2702] vs 35.8% [n = 7447]; adjusted difference, −22.4 [95% CI, −23.2 to −21.6] percentage points) (Table 2). However, at ages 20 to 21 years, when both groups were eligible for Medicaid owing to the expansion, the likelihood of being uninsured for 3 or more months was similar (21.6% [n = 4414] vs 22.0% [n = 4575]; adjusted difference, −0.1 [95% CI, −0.9 to 0.7] percentage points).

**Table 2.** Insurance Coverage Outcomes for the Preexpansion vs Postexpansion Cohorts at Ages 18 to 21 Years^a^

	Unadjusted, No. (%)		Adjusted difference, percentage points (95% CI)^b^
	Preexpansion cohort (n = 20 777)	Postexpansion cohort (n = 20 470)	
**Ages 18-19 y**			
Uninsured for ≥3 mo	4750 (22.9)	891 (4.4)	−18.4 (−19.0 to −17.7)
Medicaid for ≥3 mo	19 862 (95.6)	20 110 (98.2)	2.6 (2.2 to 2.9)
Commercial ESI for ≥3 mo	793 (3.8)	538 (2.6)	−1.2 (−1.5 to −0.8)
Commercial individual for ≥3 mo^c^	1168 (5.6)	NR^d^	−5.6 (−5.9 to −5.3)
**Ages 19-20 y**			
Uninsured for ≥3 mo	7447 (35.8)	2702 (13.2)	−22.4 (−23.2 to −21.6)
Medicaid for ≥3 mo	9574 (46.1)	17 933 (87.6)	41.7 (40.9 to 42.5)
Commercial ESI for ≥3 mo	1760 (8.5)	1070 (5.2)	−3.1 (−3.6 to −2.6)
Commercial individual for ≥3 mo^c^	3496 (16.8)	686 (3.4)	−13.5 (−14.1 to −12.9)
**Ages 20-21 y**			
Uninsured for ≥3 mo	4575 (22.0)	4414 (21.6)	−0.1 (−0.9 to 0.7)
Medicaid for ≥3 mo	14 729 (70.9)	15 054 (73.5)	2.3 (1.4 to 3.1)
Commercial ESI for ≥3 mo	2129 (10.2)	1455 (7.1)	−3.0 (−3.6 to −2.5)
Commercial individual for ≥3 mo^c^	214 (1.0)	1206 (5.9)	5.0 (4.7 to 5.4)
**Ages 18-21 y**			
Continuous insurance coverage (any type)^e^			
For ≥12 mo	13 234 (63.7)	19 272 (94.1)	30.5 (29.7 to 31.2)
For ≥24 mo	9221 (44.4)	15 868 (77.5)	33.0 (32.1 to 33.9)
Continuous Medicaid coverage^e^			
For ≥12 mo	9817 (47.2)	17 302 (84.5)	37.2 (36.4 to 38.1)
For ≥24 mo	5297 (25.5)	11 362 (55.5)	29.7 (28.8 to 30.6)

Abbreviations: ESI, employer-sponsored insurance; NR, not reported.

^a^ The preexpansion cohort included individuals who were aged 18 years and enrolled in Medicaid in January 2012; these individuals turned age 19 years before Medicaid expansion in 2014. The postexpansion cohort included individuals who were aged 18 years and enrolled in Medicaid in January 2014; these individuals turned age 19 years after Medicaid expansion.

^b^ Adjusted difference controlled for sex, low socioeconomic status zip code, percentages of races and ethnicities within a zip code (reference group: non-Hispanic or non-Latino White), and pediatric medical complexity (reference group: no chronic disease).

^c^ Classified enrollees as having commercial insurance if enrollment records overlapped with Medicaid.

^d^ Exact number was redacted in accordance with the cell size suppression policy.

^e^ Any continuous insurance and continuous Medicaid counted starting in January 2012 for the preexpansion cohort and January 2014 for the postexpansion cohort.

Individuals with mental health diagnoses at baseline were less likely than the overall cohort to be uninsured for 3 or more months before and after turning age 19 years, and differences in this insurance coverage outcome between the postexpansion and preexpansion cohorts were smaller than the overall cohort at ages 18 to 19 years (1.2% [n = 47 of 3855] vs 8.7% [n = 302 of 3454]; adjusted difference, −7.7 [95% CI, −8.7 to −6.6] percentage points) and 19 to 20 years (6.6% [n = 253] vs 18.4% [n = 637]; adjusted difference, −11.8 [95% CI, −13.3 to −10.2] percentage points) (Table 3).

**Table 3.** Insurance Coverage Outcomes for the Preexpansion vs Postexpansion Cohorts at Ages 18 to 21 Years Among Enrollees With Any Mental Health Diagnosis in Baseline Year^a^^,^^b^

	Unadjusted, No. (%)		Adjusted difference, percentage points (95% CI)^c^
	Preexpansion cohort (n = 3454)	Postexpansion cohort (n = 3855)	
**Ages 18-19 y**			
Uninsured for ≥3 mo	302 (8.7)	47 (1.2)	−7.7 (−8.7 to −6.6)
Medicaid for ≥3 mo	3377 (97.8)	3834 (99.5)	1.7 (1.1 to 2.2)
Commercial ESI for ≥3 mo	108 (3.1)	92 (2.4)	−0.8 (−1.6 to 0.0)
Commercial individual for ≥3 mo^d^	257 (7.4)	NR^e^	−7.4 (−8.3 to −6.5)
**Ages 19-20 y**			
Uninsured for ≥3 mo	637 (18.4)	253 (6.6)	−11.8 (−13.3 to −10.2)
Medicaid for ≥3 mo	2291 (66.3)	3548 (92.0)	26.4 (24.5 to 28.2)
Commercial ESI for ≥3 mo	234 (6.8)	166 (4.3)	−2.5 (−3.6 to −1.4)
Commercial individual for ≥3 mo^d^	649 (18.8)	97 (2.5)	−16.7 (−18.1 to −15.2)
**Ages 20-21 y**			
Uninsured for ≥3 mo	372 (10.8)	499 (12.9)	2.4 (0.9 to 3.9)
Medicaid for ≥3 mo	2899 (83.9)	3235 (83.9)	−0.3 (−2.1 to 1.4)
Commercial ESI for ≥3 mo	275 (8.0)	210 (5.4)	−2.5 (−3.7 to −1.3)
Commercial individual for ≥3 mo^d^	48 (1.4)	180 (4.7)	3.6 (2.8 to 4.3)
**Age 18-21 y**			
Continuous insurance coverage (any type)^f^			
For ≥12 mo	2786 (80.7)	3748 (97.2)	16.8 (15.4 to 18.3)
For ≥24 mo	2235 (64.7)	3343 (86.7)	22.3 (20.3 to 24.3)
Continuous Medicaid coverage^f^			
For ≥12 mo	2314 (67.0)	3501 (90.8)	24.3 (22.4 to 26.1)
For ≥24 mo	1579 (45.7)	2645 (68.6)	23.4 (21.1 to 25.6)

Abbreviations: ESI, employer-sponsored insurance; NR, not reported.

^a^ The preexpansion cohort included individuals who were aged 18 years and enrolled in Medicaid in January 2012; these individuals turned age 19 years before Medicaid expansion in 2014. The postexpansion cohort included individuals who were aged 18 years and enrolled in Medicaid in January 2014; these individuals turned age 19 years after Medicaid expansion.

^b^ Mental health diagnoses included anxiety disorders; depression; attention-deficit/hyperactivity disorder, conduct disorders, and hyperkinetic syndrome; bipolar disorder; schizophrenia and other psychotic disorders; or personality disorders.

^c^ Adjusted difference controlled for sex, low socioeconomic status zip code, percentages of races and ethnicities within a zip code (reference group: non-Hispanic or non-Latino White), and pediatric medical complexity (reference group: no chronic disease).

^d^ Classified enrollees as having commercial insurance if enrollment records overlapped with Medicaid.

^e^ Exact number was redacted in accordance with the cell size suppression policy.

^f^ Any continuous insurance and continuous Medicaid counted starting in January 2012 for the preexpansion cohort and January 2014 for the postexpansion cohort.

### Medicaid Coverage

As expected, nearly all individuals in both cohorts had Medicaid coverage for 3 or more months at ages 18 to 19 years. At ages 19 to 20 years, differences in this outcome between the groups were significant: 87.6% (n = 17 933) of the postexpansion vs 46.1% (n = 9574) of the preexpansion cohort had Medicaid for 3 or more months (adjusted difference, 41.7 [95% CI, 40.9-42.5] percentage points, Table 2). At ages 20 to 21 years, when both the preexpansion and postexpansion cohorts were eligible for Medicaid owing to the expansion, the differences in the likelihood of having Medicaid coverage for 3 or more months became much smaller (70.9% [n = 14 729] vs 73.5% [n = 15 054]; adjusted difference, 2.3 [95% CI, 1.4-3.1] percentage points).

### Commercial Coverage

The postexpansion cohort was less likely than the preexpansion cohort to have employer-sponsored insurance coverage for 3 or more months at ages 18 to 19 years (2.6% [n = 538 of 20 470] vs 3.8% [n = 793 of 20 777]; adjusted difference, −1.2 [95% CI, −1.5 to −0.8] percentage points), 19 to 20 years (5.2% [n = 1070] vs 8.5% [n = 1760]; adjusted difference, −3.1 [95% CI, −3.6 to −2.6] percentage points), and 20 to 21 years (7.1% [n = 1455] vs 10.2% [n = 2129]; adjusted difference, −3.0 [95% CI, −3.6 to −2.5] percentage points) (Table 2). The postexpansion cohort was also less likely to have individual commercial insurance at ages 18 to 19 years (not reported because of the small number of individuals vs 5.6% [n = 1168]; adjusted difference, −5.6 [95% CI, −5.9 to −5.3] percentage points) and 19 to 20 years (3.4% [n = 686] vs 16.8% [n = 3496]; adjusted difference, −13.5 [95% CI, −14.1 to −12.9] percentage points). The postexpansion cohort was more likely to have individual commercial insurance at ages 20 to 21 years compared with the preexpansion cohort (5.9% [n = 1206] vs 1.0% [n = 214]; adjusted difference, 5.0 [95% CI, 4.7-5.4] percentage points), consistent with the temporary Medicaid enrollments for Health Connector enrollees in 2014. Differences in coverage were similar among individuals with baseline mental health diagnoses (Table 3).

### Continuous Coverage

The postexpansion vs preexpansion cohort was significantly more likely to have continuous insurance coverage of any type for 12 or more months (94.1% [n = 19 272 of 20 470] vs 63.7% [n = 13 234 of 20 777]; adjusted difference, 30.5 [95% CI, 29.7-31.2] percentage points) and 24 or more months (77.5% [n = 15 868] vs 44.4% [n = 9221]; adjusted difference, 33.0 [95% CI, 32.1-33.9] percentage points) during the observation period (Table 2). These differences were also significant among individuals with mental health diagnoses (≥12 months: 97.2% [n = 3748 of 3855] vs 80.7% [n = 2786 of 3454]; adjusted difference, 16.8 [95% CI, 15.4-18.3] percentage points; ≥24 months: 86.7% [n = 3343] vs 64.7% [n = 2235]; adjusted difference, 22.3 [95% CI, 20.3-24.3] percentage points).

As expected, differences between the postexpansion and preexpansion cohort in the likelihood of maintaining continuous coverage were largely associated with changes in having continuous Medicaid coverage overall (≥12 months: 84.5% [n = 17 302 of 20 470] vs 47.2% [n = 9817 of 20 777]; adjusted difference, 37.2 [95% CI, 36.4-38.1] percentage points; ≥24 months: 55.5% [n = 11 362] vs 25.5% [n = 5297]; adjusted difference, 29.7 [95% CI, 28.8-30.6] percentage points; Table 2) and for those with mental health diagnoses (≥12 months: 90.8% [n = 3501 of 3855] vs 67.0% [n = 2314 of 3454]; adjusted difference, 24.3 [95% CI, 22.4-26.1] percentage points; ≥24 months: 68.6% [n = 2645] vs 45.7% [n = 1579]; adjusted difference, 23.4 [95% CI, 21.1-25.6] percentage points; Table 3).

### Sensitivity Analyses

In sensitivity analyses in which we examined insurance coverage for 6 or more months (eTables 2 and 3 in the Supplement), changed the coverage type of those with concurrent records of both commercial and Medicaid coverage to Medicaid (eTables 4 and 5 in the Supplement), and excluded self-insured commercial plans (eTables 6 and 7 in the Supplement), the results were similar to the results of the primary analysis.

## Discussion

This study found Medicaid expansion was associated with substantial reductions in disenrollment from Medicaid and the likelihood of becoming uninsured for Medicaid-enrolled children entering young adulthood. Young adults previously enrolled in Medicaid were less likely to have employer-sponsored coverage after Medicaid expansion, suggestive of crowd out (eg, individuals dropping employer-based coverage to enroll in Medicaid). After the Medicaid expansion in 2014, many enrollees who had previously aged out of Medicaid at age 19 years regained coverage. These findings were similar among enrollees with baseline mental health diagnoses.

Nationally, Medicaid expansion under the ACA substantially reduced the number of uninsured adults with low income.^2^ In this study, Medicaid expansion in Massachusetts was associated with substantial smoothing of fluctuations in insurance coverage for Medicaid enrollees turning age 19 years. For example, the proportion of previous child Medicaid enrollees being uninsured for at least 3 months at ages 18 to 19 and 19 to 20 years declined by 18 to 22 percentage points in the overall population, and the proportion with continuous Medicaid enrollment for 24 or more months during ages 18 to 21 years nearly doubled (77.5% vs 44.4%). The possibility that Medicaid expansion might have modestly crowded out employer-sponsored insurance coverage for young adults could also represent affordability gains for young adults or their families if young adults had otherwise remained the sole dependent on a parent’s employer-sponsored insurance plan. In 2014, the mean employee contribution for family coverage was $4823 compared with a household income of $35 775 for a family of 4 at 150% of the FPL.^25^

Previous research among both adults and children with Medicaid suggests that reducing gaps in coverage could be associated with reductions in acute care use, particularly for individuals with ambulatory care–sensitive conditions and chronic conditions.^26,27,28^ We found Medicaid expansion was associated with an increased likelihood of maintaining continuous Medicaid enrollment among young adults with mental health diagnoses. Medicaid coverage gains owing to the ACA have been associated with increases in receipt of mental health and substance use disorder treatment, less unmet need for care, and lower rates of suicide death, which underscores the potential clinical benefits of reducing insurance gaps among young adults with mental illness.^29,30,31^

Among those who lost Medicaid coverage at age 19 years in 2012 and 2013, many regained this coverage in 2014 after the state expanded Medicaid eligibility. Nearly three-quarters of both cohorts (70.9% in the preexpansion cohort and 73.5% in the postexpansion cohort) had Medicaid coverage for at least 3 months at ages 20 to 21 years, when both groups were eligible for Medicaid owing to expansion. Nevertheless, approximately 20% overall were uninsured for at least 3 months at ages 20 to 21 years. Massachusetts likely represents a favorable scenario for young adult coverage losses given the state’s uninsurance rate of less than 5%,^32^ which is the lowest in the nation, and the state’s relatively longer experience with insurance reforms similar to the ACA. It is possible that administrative burdens associated with newly qualifying for Medicaid as an adult were a barrier to enrollment as individuals turned age 21 years. Some families with Medicaid in Massachusetts are eligible for streamlined annual redeterminations, including those with incomes at or below 150% of the FPL and those with enrolled children receiving Supplemental Nutrition Assistance Program benefits, which could have helped some young adults aged 19 to 20 years maintain continuous Medicaid coverage as children.^33^

### Limitations

This study has limitations. Because the study period included 2014, the year the Massachusetts Health Connector experienced technical difficulties and all applicants were temporarily enrolled in Medicaid, we were unable to reliably quantify differences in the likelihood of having individual commercial coverage. Approximately 17% of the preexpansion cohort had 3 or more months of individual commercial insurance at ages 19 to 20 years, however, likely owing to the availability of subsidies for consumers with a low income.

We also could not identify those who moved out of state or who died outside the hospital. We lacked information at the individual level on disability status, household income, or citizenship and residency information to ascertain whether individuals were eligible for Medicaid in each period. Some employers stopped contributing data on self-insured plans starting in 2016; thus, we could overestimate uninsured rates in 2016 for individuals covered by such plans. In addition, we focused on changes in insurance coverage among young adults previously enrolled in Medicaid as children and did not examine the implications of Medicaid expansion for Medicaid enrollment among all eligible young adults in Massachusetts.

## Conclusions

In this cohort study of young adults, Medicaid expansion reforms in Massachusetts were associated with substantial reductions in the likelihood of becoming uninsured for child Medicaid enrollees entering adulthood.

## Notes

Supplement. **eFigure.** Diagram of Study Cohorts and Follow-up Periods **eTable 1.** Study Population Baseline-Year Characteristics: Medicaid Enrollees Aged 18 Years in January 2012 (Before Expansion) or January 2014 (After Expansion) With Any Mental Health Diagnosis in Baseline Year **eTable 2.** Sensitivity Analysis: Insurance Coverage Outcomes for the Pre- vs Postexpansion Cohorts at Ages 18 to 21 Years Using 6-Month Cut-off **eTable 3.** Sensitivity Analysis: Insurance Coverage Outcomes for the Pre- vs Postexpansion Cohorts at Ages 18 to 21 Years Using 6-Month Cut-off Among Enrollees With Any Mental Health Diagnoses in Baseline Year **eTable 4.** Sensitivity Analysis: Insurance Coverage Outcomes for the Pre- vs Postexpansion Cohorts at Ages 18 to 21 Years Classifying Enrollment as Medicaid if Overlapping Records Indicated Commercial ESI or Commercial Individual Insurance **eTable 5.** Sensitivity Analysis: Insurance Coverage Outcomes for the Pre- vs Postexpansion Cohorts at Ages 18 to 21 Years Classifying Enrollment as Medicaid if Overlapping Records Indicated Commercial ESI or Commercial Individual Insurance Among Enrollees With Any Mental Health Diagnoses in Baseline Year **eTable 6.** Sensitivity Analysis: Insurance Coverage Outcomes for the Pre- vs Postexpansion Cohorts at Ages 20 to 21 Years, Excluding Self-insured Commercial Plans **eTable 7.** Sensitivity Analysis: Insurance Coverage Outcomes for the Pre- vs Postexpansion Cohorts at Ages 20 to 21 Years Among Enrollees With Any Mental Health Diagnoses in Baseline Year, Excluding Self-insured Commercial Plans
