# Supplementary material for: Changes in Insurance Coverage Continuity After Affordable Care Act Expansion of Medicaid Eligibility for Young Adults With Low Income in Massachusetts
Source: JAMA Health Forum. 2022 Jul 15;3(7):e221996. doi: 10.1001/jamahealthforum.2022.1996 (PMC9287752; doi:10.1001/jamahealthforum.2022.1996)
Supplement: Supplement. — eFigure. Diagram of Study Cohorts and Follow-up Periods eTable 1. Study Population Baseline-Year Characteristics: Medicaid Enrollees Aged 18 Years in January 2012 (Before Expansion) or January 2014 (After Expansion) With Any Mental Health Diagnosis in Baseline Year eTable 2. Sensitivity Analysis: Insurance Coverage Outcomes for the Pre- vs Postexpansion Cohorts at Ages 18 to 21 Years Using 6-Month Cut-off eTable 3. Sensitivity Analysis: Insurance Coverage Outcomes for the Pre- vs Postexpansion Cohorts at Ages 18 to 21 Years Using 6-Month Cut-off Among Enrollees With Any Mental Health Diagnoses in Baseline Year eTable 4. Sensitivity Analysis: Insurance Coverage Outcomes for the Pre- vs Postexpansion Cohorts at Ages 18 to 21 Years Classifying Enrollment as Medicaid if Overlapping Records Indicated Commercial ESI or Commercial Individual Insurance eTable 5. Sensitivity Analysis: Insurance Coverage Outcomes for the Pre- vs Postexpansion Cohorts at Ages 18 to 21 Years Classifying Enrollment as Medicaid if Overlapping Records Indicated Commercial ESI or Commercial Individual Insurance Among Enrollees With Any Mental Health Diagnoses in Baseline Year eTable 6. Sensitivity Analysis: Insurance Coverage Outcomes for the Pre- vs Postexpansion Cohorts at Ages 20 to 21 Years, Excluding Self-insured Commercial Plans eTable 7. Sensitivity Analysis: Insurance Coverage Outcomes for the Pre- vs Postexpansion Cohorts at Ages 20 to 21 Years Among Enrollees With Any Mental Health Diagnoses in Baseline Year, Excluding Self-insured Commercial Plans [file jamahealthforum-e221996-s001.pdf]

Supplementary Online Content

Fung V, Yang Z, Cook BL, Hsu J, Newhouse JP. Changes in insurance coverage continuity after Affordable Care Act expansion of Medicaid eligibility for young adults with low income in Massachusetts. *JAMA Health Forum*. 2022:3(7):e221996. doi:10.1001/jamahealthforum.2022.1996

eFigure. Diagram of Study Cohorts and Follow-up Periods
eTable 1. Study Population Baseline-Year Characteristics: Medicaid Enrollees Aged 18 Years in January 2012 (Before Expansion) or January 2014 (After Expansion) With Any Mental Health Diagnosis in Baseline Year
eTable 2. Sensitivity Analysis: Insurance Coverage Outcomes for the Pre- vs Postexpansion Cohorts at Ages 18 to 21 Years Using 6-Month Cut-off
eTable 3. Sensitivity Analysis: Insurance Coverage Outcomes for the Pre- vs Postexpansion Cohorts at Ages 18 to 21 Years Using 6-Month Cut-off Among Enrollees With Any Mental Health Diagnoses in Baseline Year
eTable 4. Sensitivity Analysis: Insurance Coverage Outcomes for the Pre- vs Postexpansion Cohorts at Ages 18 to 21 Years Classifying Enrollment as Medicaid if Overlapping Records Indicated Commercial ESI or Commercial Individual Insurance
eTable 5. Sensitivity Analysis: Insurance Coverage Outcomes for the Pre- vs Postexpansion Cohorts at Ages 18 to 21 Years Classifying Enrollment as Medicaid if Overlapping Records Indicated Commercial ESI or Commercial Individual Insurance Among Enrollees With Any Mental Health Diagnoses in Baseline Year
eTable 6. Sensitivity Analysis: Insurance Coverage Outcomes for the Pre- vs Postexpansion Cohorts at Ages 20 to 21 Years, Excluding Self-insured Commercial Plans
eTable 7. Sensitivity Analysis: Insurance Coverage Outcomes for the Pre- vs Postexpansion Cohorts at Ages 20 to 21 Years Among Enrollees With Any Mental Health Diagnoses in Baseline Year, Excluding Self-insured Commercial Plans

This supplementary material has been provided by the authors to give readers additional information about their work.

© 2022 Fung V et al. *JAMA Health Forum.*

eFigure. Diagram of study cohorts and follow-up periods

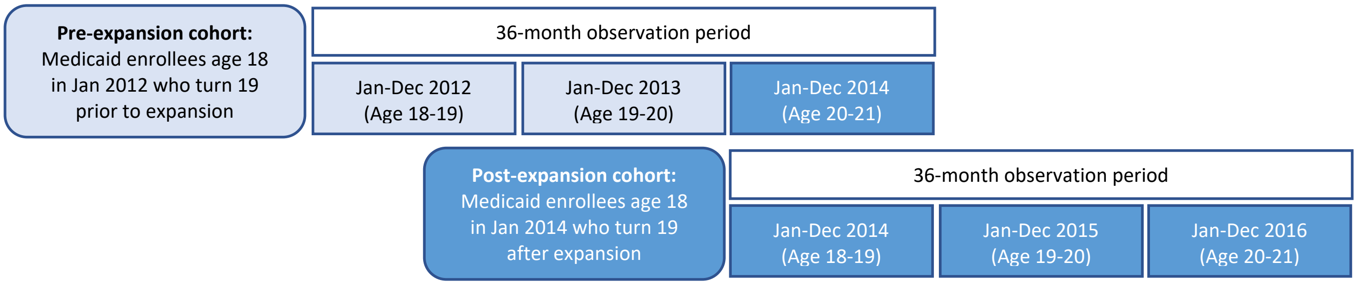

Notes: Dark blue boxes indicate calendar years after Massachusetts expanded Medicaid to adults with incomes up to 133% of the federal poverty level (FPL) and Medicaid eligibility as a child to 19 and 20-year-olds with incomes up to 150% FPL.

© 2022 Fung V et al. *JAMA Health Forum.*

eTable 1. Study population baseline-year characteristics: Medicaid enrollees aged 18 years in January 2012 (before expansion) or January 2014 (after expansion) with any mental health diagnosis in baseline year

Characteristic	Pre-expansion cohort (N = 3,454)	Post-expansion cohort (N = 3,855)	p-value
**Gender, N (%):**			
Female	2,010 (58.2%)	2,224 (57.7%)	0.65
Male	1,400 (>40.5%)	1,600 (>41.5%)	
**SES:** Low SES ZIP code, N (%)	881 (25.5%)	981 (25.4%)	0.55
**Race/ethnicity:** percentage in ZIP code, mean (SD)			
Hispanic/Latino	16.7 (18.4)	17.3 (19.1)	0.20
Non-Hispanic/Latino:			
Asian	4.3 (5.3)	4.6 (5.6)	0.04
Black/African American	9.7 (14.2)	9.4 (13.9)	0.51
Other	2.9 (2.3)	3.1 (2.3)	<0.001
White	66.5 (25.9)	65.6 (26.1)	0.18
**Pediatric Medical Complexity, N (%):**			
Complex chronic disease	1,056 (30.6%)	1,200 (31.1%)	0.38
Non-complex chronic disease	1,525 (44.2%)	1,735 (45.0%)	
Without chronic disease	873 (25.3%)	920 (23.9%)	
**Current year mental health diagnoses, N (%):**			
Anxiety disorders	1,736 (50.3%)	2,104 (54.6%)	<0.001
Depression	1,643 (47.6%)	1,809 (46.9%)	0.58
ADHD, conduct disorders, and hyperkinetic syndrome	1,140 (33.0%)	1,258 (32.6%)	0.74
Bipolar disorder	826 (23.9%)	891 (23.1%)	0.42
Schizophrenia and other psychotic disorders	329 (9.5%)	353 (9.2%)	0.59
Personality disorders	64 (1.9%)	75 (1.9%)	0.77
**Mean (SD) insured months in baseline year**	11.3 (1.7)	11.8 (1.0)	<0.001

Notes: (1) Low socioeconomic status (SES) ZIP codes: >20% of households below poverty or >25% of residents ages 25 and over with less than a high school education. (2) <1% of both the pre- and post-expansion cohorts had missing/conflicting gender information (exact percentage of Males and individuals with missing/conflicting gender information redacted due to cell size suppression policy); 6.6% and 4.6% of the pre- and post-expansion cohorts had missing/conflicting SES information, respectively; 0.3% and 0.4% of the pre- and post-expansion cohorts had missing race information, respectively. (3) Robust standard errors used to calculate p-values from t-tests, except for Pediatric Medical Complexity (p-value from a chi-square test).

© 2022 Fung V et al. *JAMA Health Forum.*

eTable 2. Sensitivity analysis: Insurance coverage outcomes for the pre- vs postexpansion cohorts at ages 18 to 21 years using 6-month cut-off

	Pre-expansion cohort (N = 20,777)	Post-expansion cohort (N = 20,470)	Adjusted difference
**Ages 18 – 19:**	**N (%), Unadj.**	**N (%), Unadj.**	**Pct pts (95% CI)**
Uninsured for ≥6 months	2,335 (11.2%)	590 (2.9%)	-8.2 (-8.7, -7.7)
Medicaid for ≥6 months	16,898 (81.3%)	19,336 (94.5%)	12.9 (12.3, 13.6)
Commercial ESI for ≥6 months	298 (1.4%)	291 (1.4%)	0.0 (-0.3, 0.2)
Commercial individual for ≥6 months	537 (2.6%)	Not reported	-2.6 (-2.8, -2.4)
**Ages 19 – 20:**			
Uninsured for ≥6 months	5,431 (26.1%)	1,839 (9.0%)	-17.1 (-17.8, -16.4)
Medicaid for ≥6 months	8,332 (40.1%)	15,722 (76.8%)	36.8 (35.9, 37.7)
Commercial ESI for ≥6 months	1,254 (6.0%)	774 (3.8%)	-2.2 (-2.6, -1.8)
Commercial individual for ≥6 months	2,620 (12.6%)	484 (2.4%)	-10.3 (-10.8, -9.8)
**Ages 20 – 21:**			
Uninsured for ≥6 months	3,872 (18.6%)	3,351 (16.4%)	-2.0 (-2.7, -1.2)
Medicaid for ≥6 months	13,498 (65.0%)	14,108 (68.9%)	3.6 (2.7, 4.5)
Commercial ESI for ≥6 months	1,817 (8.7%)	973 (4.8%)	-3.9 (-4.4, -3.4)
Commercial individual for ≥6 months	154 (0.7%)	881 (4.3%)	3.7 (3.4, 4.0)

Notes: (1) Adjusted difference controlling for gender, low SES ZIP code, percentages of race/ethnicity within ZIP code (reference group: non-Hispanic/Latino White), and Pediatric Medical Complexity (reference group: without chronic disease). (2) Robust standard errors used to calculate p-values. (3) Classified enrollees as having commercial insurance if enrollment records overlapped with Medicaid. Exact number of individuals in post-expansion cohort with commercial insurance for ≥6 months was redacted due to cell size suppression policy.

© 2022 Fung V et al. *JAMA Health Forum.*

eTable 3. Sensitivity analysis: Insurance coverage outcomes for the pre- vs postexpansion cohorts at ages 18 to 21 years using 6-month cut-off among enrollees with any mental health diagnoses in baseline year

	**Pre-expansion cohort (N = 3,454)**	**Post-expansion cohort (N = 3,855)**	**Adjusted difference Pct pts (95% CI)**
**Ages 18 – 19:**	**N (%), Unadj.**	**N (%), Unadj.**	
Uninsured for ≥6 months	82 (2.4%)	24 (0.6%)	-1.7 (-2.3, -1.2)
Medicaid for ≥6 months	3,136 (90.8%)	3,766 (97.7%)	7.1 (6.0, 8.2)
Commercial ESI for ≥6 months	53 (1.5%)	59 (1.5%)	0.0 (-0.6, 0.6)
Commercial individual for ≥6 months	130 (3.8%)	0 (0.0%)	-3.9 (-4.5, -3.2)
**Ages 19 – 20:**			
Uninsured for ≥6 months	378 (10.9%)	160 (4.2%)	-6.7 (-8.0, -5.5)
Medicaid for ≥6 months	2,084 (60.3%)	3,296 (85.5%)	25.8 (23.7, 27.8)
Commercial ESI for ≥6 months	163 (4.7%)	124 (3.2%)	-1.6 (-2.6, -0.7)
Commercial individual for ≥6 months	501 (14.5%)	70 (1.8%)	-13.0 (-14.3, -11.7)
**Ages 20 – 21:**			
Uninsured for ≥6 months	281 (8.1%)	341 (8.8%)	0.9 (-0.4, 2.2)
Medicaid for ≥6 months	2,780 (80.5%)	3,090 (80.2%)	-0.7 (-2.6, 1.2)
Commercial ESI for ≥6 months	232 (6.7%)	142 (3.7%)	-3.1 (-4.1, -2.0)
Commercial individual for ≥6 months	44 (1.3%)	133 (3.5%)	2.5 (1.7, 3.2)

Notes: (1) Adjusted difference controlling for gender, low SES ZIP code, percentages of race/ethnicity within ZIP code (reference group: non-Hispanic/Latino White), and Pediatric Medical Complexity (reference group: without chronic disease). (2) Robust standard errors used to calculate p-values. (3) Classified enrollees as having commercial insurance if enrollment records overlapped with Medicaid.

© 2022 Fung V et al. *JAMA Health Forum.*

eTable 4. Sensitivity analysis: Insurance coverage outcomes for the pre- vs postexpansion cohorts at ages 18 to 21 years classifying enrollment as Medicaid if overlapping records indicated commercial ESI or commercial individual insurance

	**Pre-expansion cohort (N = 20,777)**	**Post-expansion cohort (N = 20,470)**	**Adjusted difference Pct pts (95% CI)**
**Ages 18 – 19:**	**N (%), Unadj.**	**N (%), Unadj.**	
Medicaid for ≥3 months	20,033 (96.4%)	20,185 (98.6%)	2.1 (1.8, 2.4)
Commercial ESI for ≥3 months	463 (2.2%)	103 (0.5%)	-1.7 (-1.9, -1.5)
Commercial individual for ≥3 months	819 (3.9%)	Not reported	-3.9 (-4.2, -3.7)
**Ages 19 – 20:**			
Medicaid for ≥3 months	9,977 (48.0%)	18,557 (90.7%)	42.9 (42.1, 43.7)
Commercial ESI for ≥3 months	1,544 (7.4%)	628 (3.1%)	-4.3 (-4.7, -3.8)
Commercial individual for ≥3 months	3,190 (15.4%)	576 (2.8%)	-12.6 (-13.2, -12.1)
**Ages 20 – 21:**			
Medicaid for ≥3 months	15,345 (73.9%)	15,404 (75.3%)	1.0 (0.1, 1.8)
Commercial ESI for ≥3 months	1,180 (5.7%)	863 (4.2%)	-1.4 (-1.8, -0.9)
Commercial individual for ≥3 months	151 (0.7%)	1,047 (5.1%)	4.5 (4.2, 4.9)
**Ages 18 – 21:**			
Continuous Medicaid for ≥12 months	10,316 (49.7%)	17,774 (86.8%)	37.1 (36.3, 37.9)
Continuous Medicaid for ≥24 months	5,658 (27.2%)	11,873 (58.0%)	30.5 (29.6, 31.4)

Notes: (1) Adjusted difference controlling for gender, low SES ZIP code, percentages of race/ethnicity within ZIP code (reference group: non-Hispanic/Latino White), and Pediatric Medical Complexity (reference group: without chronic disease). (2) Robust standard errors used to calculate p-values. (3) Classified enrollees as having Medicaid insurance if enrollment records overlapped with commercial ESI or commercial individual.
(4) Continuous Medicaid counted starting in Jan. 2012 and 2014 for pre- and post-expansion cohort, respectively. Exact number of individuals in post-expansion cohort with commercial insurance for ≥6 months was redacted due to cell size suppression policy.

eTable 5. Sensitivity analysis: Insurance coverage outcomes for the pre- vs postexpansion cohorts at ages 18 to 21 years classifying enrollment as Medicaid if overlapping records indicated commercial ESI or commercial individual insurance among enrollees with any mental health diagnosis in baseline year

	**Pre-expansion cohort (N = 3,454)**	**Post-expansion cohort (N = 3,855)**	**Adjusted difference Pct pts (95% CI)**
**Ages 18 – 19:**	**N (%), Unadj.**	**N (%), Unadj.**	
Medicaid for ≥3 months	3,414 (98.8%)	3,850 (99.9%)	1.0 (0.7, 1.4)
Commercial ESI for ≥3 months	49 (1.4%)	15 (0.4%)	-1.0 (-1.5, -0.6)
Commercial individual for ≥3 months	167 (4.8%)	0 (0.0%)	-5.0 (-5.8, -4.3)
**Ages 19 – 20:**			
Medicaid for ≥3 months	2,382 (69.0%)	3,644 (94.5%)	26.3 (24.5, 28.0)
Commercial ESI for ≥3 months	195 (5.6%)	90 (2.3%)	-3.3 (-4.3, -2.4)
Commercial individual for ≥3 months	563 (16.3%)	79 (2.0%)	-14.7 (-16.1, -13.4)
**Ages 20 – 21:**			
Medicaid for ≥3 months	3,025 (87.6%)	3,288 (85.3%)	-2.6 (-4.3, -1.0)
Commercial ESI for ≥3 months	122 (3.5%)	115 (3.0%)	-0.6 (-1.4, 0.3)
Commercial individual for ≥3 months	19 (0.6%)	151 (3.9%)	3.5 (2.9, 4.2)
**Ages 18 – 21:**			
Continuous Medicaid for ≥12 months	2,448 (70.9%)	3,587 (93.0%)	22.7 (20.9, 24.4)
Continuous Medicaid for ≥24 months	1,700 (49.2%)	2,753 (71.4%)	22.7 (20.5, 24.9)

Notes: (1) Adjusted difference controlling for gender, low SES ZIP code, percentages of race/ethnicity within ZIP code (reference group: non-Hispanic/Latino White), and Pediatric Medical Complexity (reference group: without chronic disease). (2) Robust standard errors used to calculate p-values. (3) Classified enrollees as having Medicaid insurance if enrollment records overlapped with commercial ESI or commercial individual.
(4) Continuous Medicaid counted starting in Jan. 2012 and 2014 for pre- and post-expansion cohort, respectively.

© 2022 Fung V et al. *JAMA Health Forum.*

eTable 6. Sensitivity analysis: Insurance coverage outcomes for the pre- vs postexpansion cohorts at ages 20 to 21 years, excluding self-insured commercial plans

	Pre-expansion cohort (N = 20,777)	Post-expansion cohort (N = 20,470)	Adjusted difference Pct pts (95% CI)
**Ages 20 – 21:**	**N (%), Unadj.**	**N (%), Unadj.**	
Uninsured for ≥3 months	4,970 (23.9%)	4,550 (22.2%)	-1.3 (-2.1, -0.5)
Medicaid for ≥3 months	15,003 (72.2%)	15,159 (74.1%)	1.4 (0.6, 2.3)
Commercial ESI for ≥3 months	1,270 (6.1%)	1,162 (5.7%)	-0.3 (-0.8, 0.1)
Commercial individual for ≥3 months	217 (1.0%)	1,214 (5.9%)	5.1 (4.7, 5.4)

Notes: (1) Adjusted difference controlling for gender, low SES ZIP code, percentages of race/ethnicity within ZIP code (reference group: non-Hispanic/Latino White), and Pediatric Medical Complexity (reference group: without chronic disease). (2) Robust standard errors used to calculate p-values. (3) Classified enrollees as having commercial insurance if enrollment records overlapped with Medicaid. (4) Commercial ESI does not include self-insured plans.

© 2022 Fung V et al. *JAMA Health Forum.*

eTable 7. Sensitivity analysis: Insurance coverage outcomes for the pre- vs postexpansion cohorts at ages 20 to 21 years among enrollees with any mental health diagnosis in baseline year, excluding self-insured commercial plans

	**Pre-expansion cohort (N = 3,454)**	**Post-expansion cohort (N = 3,855)**	**Adjusted difference Pct pts (95% CI)**
**Ages 20 – 21:**	**N (%), Unadj.**	**N (%), Unadj.**	
Uninsured for ≥3 months	414 (12.0%)	518 (13.4%)	1.7 (0.1, 3.3)
Medicaid for ≥3 months	2,943 (85.2%)	3,251 (84.3%)	-1.2 (-2.9, 0.5)
Commercial ESI for ≥3 months	170 (4.9%)	164 (4.3%)	-0.7 (-1.7, 0.3)
Commercial individual for ≥3 months	48 (1.4%)	181 (4.7%)	3.6 (2.8, 4.4)

Notes: (1) Adjusted difference controlling for gender, low SES ZIP code, percentages of race/ethnicity within ZIP code (reference group: non-Hispanic/Latino White), and Pediatric Medical Complexity (reference group: without chronic disease). (2) Robust standard errors used to calculate p-values. (3) Classified enrollees as having commercial insurance if enrollment records overlapped with Medicaid. (4) Commercial ESI does not include self-insured plans.

© 2022 Fung V et al. *JAMA Health Forum.*
